# A patient perspective on applying intermittent fasting in gynecologic cancer

**DOI:** 10.1186/s13104-023-06453-5

**Published:** 2023-08-29

**Authors:** Ashley Redding, Sara Santarossa, Dana Murphy, Mary Priyanka Udumula, Adnan Munkarah, Miriana Hijaz, Ramandeep Rattan

**Affiliations:** 1 Public Health Sciences, Henry Ford Health, Detroit, MI USA; 2 Department of Obstetrics, Gynecology and Reproductive Biology, College of Human Medicine, Michigan State University, East Lansing, USA; 3 Division of Gynecology Oncology, Women’s Health Sciences, Henry Ford Health, Detroit, MI USA; 4 Department of Oncology, Wayne State University, Detroit, MI 48202 USA

**Keywords:** Intermittent fasting, Patient perspectives, Randomized control trial, Feasibility

## Abstract

**Objective:**

Researchers sought patient feedback on a proposed randomized controlled trial (RCT) in which gynecological cancer patients would modify their diets with intermittent fasting to gain insight into patients’ perspectives, receptivity, and potential obstacles. A convenience sample of 47 patients who met the inclusion criteria of the proposed RCT provided their feedback on the feasibility and protocols of the RCT using a multi-method approach consisting of focus groups (n = 8 patients) and surveys (n = 36 patients).

**Results:**

Patients were generally receptive to the concept of intermittent fasting, and many expressed an interest in attempting it themselves. Patients agreed that the study design was feasible in terms of study assessments, clinic visits, and biospecimen collection. Feedback on what could facilitate adherence included convenient appointment scheduling times and the availability of the research team to answer questions. Regarding recruitment, patients offered suggestions for study advertisements, with the majority concurring that a medical professional approaching them would increase their likelihood of participation.

**Supplementary Information:**

The online version contains supplementary material available at 10.1186/s13104-023-06453-5.

## Introduction

As the research community undergoes a “revolutionary” paradigm shift from viewing patients as “subjects” to “experts,” new projects can involve patients in the research lifecycle, from study design to data translation [1–3]. Patients are responsible for many developments of interest, such as identifying research priorities [3], and helping recruit and retain participants [4–6]. Patients benefit via new access to scientific information, a place to process their experiences, and a sense of purpose from making a difference [7].

The present study leverages lived experiences of current and former gynecologic cancer patients to understand the feasibility and compliance concerns of a proposed randomized control trial (RCT), Intermittent Fasting to Restrict Cancer (iFIRE-C), in which gynecological cancer patients in remission or undergoing chemotherapy/radiation treatment will be randomly assigned to eat regularly (control group) or perform intermittent fasting (IF).

## Methods

### Strategy

A mixed-methods strategy involving focus groups (FGs) and a brief online survey was employed to gain patient perspectives. The Institutional Review Board of Henry Ford Health (HFH) granted ethical approval (#15521-31) for this study.

### Recruitment of participants

The sample pool consisted of HFH patients 18 years of age or older who currently have or previously had gynecologic cancer such as cervical, ovarian, uterine, vaginal, and vulvar. Potential participants were ascertained using a convenience sample of patients from HFH’s electronic medical records system, and a targeted email list of gynecological oncology patients at HFH’s Cancer Center. Nonprobability purposeful snowball sampling [8] was also used by HFH’s Patient Engaged Research Center [9] to identify potential participants. Potential participants received recruitment assets via email. Interested and eligible participants received a digital consent form via REDCap (Vanderbilt University, Nashville, TN) [2, 10]. Prior to the FG, consented participants were emailed iFIRE-C’s purpose, duration, procedures, and the “Fasting & Food Log.”

To collect additional perspectives, the Patient Engaged Research Center staff emailed prospective patients, excluding those who had already participated in FGs, to complete a brief online survey.

### Data generation

#### Focus groups

Virtual FGs were conducted via Webex (Cisco Systems, Inc., San Jose, CA); consent was reconfirmed. FGs were facilitated by Patient Engaged Research Center staff trained in qualitative research methodologies. A four-section moderator guide [11] was utilized. In section one, the facilitator and patients introduced themselves. The second section described the goal of gaining the patient perspective on iFIRE-C. Section three contained key questions regarding dieting experiences, opinions on IF, the study title and design, and recruitment. In the fourth section, the facilitator summarized the discussion and asked patients for further clarifications or outstanding questions. Patients were compensated for their time.

#### Survey

Similar queries as in the FGs were posed in a 24-question survey developed by investigators for this study specifically, including a mix of open- and closed-ended questions about recruitment methods, communication preferences, and intervention feasibility for iFIRE-C (Supplementary file).

### Data analysis

#### Focus groups

The FG transcripts were analyzed using deductive coding, with initial codes developed based on aspects of iFIRE-C and the moderator guide’s main questions, resulting in 6 topic areas (Table 1). This deductive analysis conceptualized patients’ perspectives across the FGs regarding the feasibility of the proposed study components, within the context of their lived cancer experiences.

**Table 1:** Topic areas resulting from deductive coding in focus groups gauging patient perspectives on iFIRE-C

Topic Area	
Patients’ past dieting experiences	
Patients’ understanding and attitude towards IF	
Thoughts on the proposed RCT study title (iFIRE-C)	
Thoughts on iFIRE-C design and assessments	
Thoughts on iFIRE-C supports	
Patient ideas for iFIRE-C recruitment and advertising	

IF, intermittent fasting; iFIRE-C, Intermittent Fasting to Restrict Cancer; RCT, randomized control trial

#### Survey

The survey data was analyzed using SPSS Statistics (Version 26; IBM, Armonk, NY). Descriptive statistics were generated for all closed-ended questions, and open-ended questions were summarized qualitatively.

## Results

### Focus Groups

After 5 weeks of advertising, 20 individuals responded, but not all signed a consent form. Eventually, 8 individuals participated across 3 FGs between June 20, 2022 and July 6, 2022. FG size ranged from 1 to 4 and lasted between 20 and 50 min. Results are presented by topic area below.

#### Patients’ past experiences with dieting

Patients had attempted WeightWatchers (WW International, New York, NY) (n = 4), IF (n = 3), and ketogenic diet (n = 1), with varying degrees of success. One individual who practices IF has been successfully maintaining it for the past 2–3 years, whereas the individual who tried a ketogenic diet could not sustain the restrictions.

#### Patients’ understanding and attitude toward IF

Most patients knew about IF and knew someone who had tried it. Regarding their perspectives on IF as cancer patients, 2 patients mentioned asking their respective doctors about IF recently, and while both were told that more scientific evidence is required, one patient stated, “*I would do it in a heartbeat if I knew it would be helpful either for survival statistics or feeling better*.” Other positive emotions centered around weight management and long-term health.

Some patients explained that receiving chemotherapy would be a barrier. Concerns included needing to take advantage of opportunities to keep food down and losing too much weight, and facilitators included the sometimes irregular “schedule” of life during treatment.

One response addressed the “information overload” experienced by cancer patients, illustrated by the quote, *“As a cancer patient, you have so much getting thrown at you…so many treatment options and doctor’s appointments and other worries, work and family and things like that. I don’t know if someone presented me with an intermittent fasting diet, like, why would you want to put me on a diet? I’m going through cancer. It would have to be explained in such a way to show me the benefits of how is this helpful to me as someone who is going through cancer treatment.”*

When asked how long patients might be able to fast, most responses ranged between 8 and 12 h. The primary concern about adherence was social relationships: eating with loved ones at home and outside. Holidays and vacations were of special concern (n = 3 mentions).

#### Thoughts on the proposed RCT title (iFIRE-C)

Participants liked the study moniker, iFIRE-C, calling it “*catchy*” (n = 5) and “*easy to understand*,” and agreed they would not alter it. One person stated that the title evokes action, and “*doing something positive*.” Another person said they were a bit “*hung up on*” the word restrict, wondering if it referred to restricting active cancer or cancer in remission.

*Thoughts on iFIRE-C design and assessments*: The patients reviewed relevant documents prior to the FGs and provided input on aspects of iFIRE-C’s RCT design. Their perspectives are outlined in Table 2.

**Table 2:** Patient perspective on iFIRE-C study assessments

iFIRE-C Study Assessment	Patient Perspective from FGs
Gradual increase of fasting period	• Thoughts on how adherence might depend on types of drinks or liquids that could be consumed during the fasting period. • Appreciation for the gradual approach that allowed them to “mentally acclimate” • Consideration for prospective participants’ work schedules (i.e., whether those who work night shift could still participate)
Commitment to 4 clinic visits	• Consensus about the amount being feasible • Convenience was most salient code (e.g. convenience of clinic location relative to potential participants’ homes, convenience of available appointment times, and convenience for caregivers, as some may have to drive potential participants to appointments) • Consensus that visits should be shorter than one hour
Biospecimens to be collected (blood, urine, and stool)	• Patients receptive to collecting samples at home, however one patient clarified that if they resided close to a clinic, it might be easier to drive there and receive clinic staff assistance with collection • Consensus that collection is feasible if supplies and clear instructions are provided • Some discomfort with stool collection, but not enough to prevent participation

FG, focus group; iFIRE-C = Intermittent Fasting to Restrict Cancer

#### Thoughts on the iFIRE-C supports

Here, patients focused on engagement with study team members, nutrition support, and compensation. Patients indicated that supports required to remain engaged in the study may differ by person. Calls, emails, and text messages were mentioned as methods to check in, and patients considered it important to use a tailored approach based on individual preference. Additionally, patients agreed that having study support available to address patient questions would be advantageous.

The moderator inquired if nutrition support from a registered dietician would encourage study compliance. Patients liked the concept, with one patient expanding that the “*general population doesn’t know the best foods, the healthiest ones that keep you full longer, or foods to eat at certain times of the day.“*

Feedback on iFIRE-C’s gratuity of $50 per 45–60-minute visit/sample collection included references to currently high gas prices and the necessity to travel to study appointments. A patient explained that while this amount would not motivate an uninterested person, cancer patients and survivors would be motivated by the possibility of improving their own condition or the condition of future patients.

*Patient suggestions foriFIRE-Crecruitment and advertisement*: Patients concurred that they would be most likely to participate if approached by a medical professional. Patient recruitment ideas included having someone present at a cancer support group and contacting people/leaving them with a callback number.

The patients also provided feedback on the drafted study flyer. They suggested flyers should include a catchy tagline requesting assistance and improve aesthetics by minimizing text. Patients believed that highlighting the benefits of participation and providing straightforward communication regarding time commitment would be relevant additions. They supported the use of both electronic and paper flyers.

### Survey

All 36 respondents were female, and the majority identified as white race. They were primarily not Hispanic or Latino, and the majority (n = 28) were 45 years of age or older.

Patients reported being most receptive to emails (n = 16), texts (n = 12), or in-person interventions (n = 13), and less receptive to telephone calls and social media approaches. The in-person approach by a medical professional would increase their likelihood of enrolling (n = 26).

Concerning communication with the study team, patients favored email (n = 15), and many were willing to receiving weekly (n = 9) or bi-monthly (n = 16) communications.

To determine the feasibility of iFIRE-C, present eating habits were assessed. Most patients endorsed eating 2–3 times (n = 21) or 4–5 times (n = 12) per day. There were mixed responses regarding nausea during chemotherapy (14 had nausea; 13 said they did not, and 9 did not respond). A total of 15 patients responded to a free-text query about the side effects of chemotherapy on their appetite. The responses centered on alterations in taste (food tastes “*like metal*”), a lack of appetite, and nausea. Regarding fasting, 23 patients had previous personal experience with fasting, and 25 indicated that their work/lifestyle would not interfere with their ability to adhere to a fasting regimen. Many patients (n = 19) agreed that they would be more likely to adhere to a fasting diet if they were permitted to consume water, coffee, or herbal tea (sans sugar/artificial sweeteners) during their fasting period. Finally, when asked if they would be interested in participating in a study doing IF for 12 h or more/day over a span of 12 + weeks, 16 patients indicated they would be interested.

## Discussion

This study aimed to evaluate the feasibility of iFIRE-C, with the goal of incorporating patient feedback. Key patient perspectives were gathered in a multi-pronged approach, which included virtual FGs and an online survey. Overall, our analyses show that the patients were receptive to the concept of IF if it would help the disease outcome.

A recent review notes that various short-term RCTs have shown that fasting may impact cancer risk factors, but long-term trials have not shown significant improvements, and further research is required [12]. Thus, given the paucity of research on IF in cancer prevention, and the importance of including patient perspective in RCT design, iFIRE-C seeks to fill a critical gap in cancer clinical trial research.

Cancer is associated with emotional distress [13–15], which may be caused by fear of recurrence, lifestyle changes, and familial and financial concern. Patients had reported their primary concerns peaked at 6 months after the cancer diagnosis and centered on their illness, treatment, and the potential outcomes of their illness [14]. In the present study, it was evident that patients, at various stages of post-diagnosis and treatment, remained concerned about cancer prevention in the future, not only for themselves, but also for others with cancer, highlighting the altruistic attitudes of the participants.

This study revealed that patients had tried diet modifications in the past, and all had some knowledge about IF and would be willing to try it. They perceived a clinical trial in that area as feasible and appeared interested in fasting for weight management. Patients deemed the proposed study design to be feasible as well. Notably, the gradual increase of the fasting window and the availability of support were viewed as especially positive. Feedback regarding the study assessments centered on the convenience of all aspects of clinic visits and sample collections. This is consistent with previous research suggesting that patient convenience can support intervention adherence [16–18].

Regarding recruitment, both FG participants and survey respondents appreciated being approached by a medical professional. Previous research has emphasized the importance of the patient-provider relationship in recruitment [19], and communication between patient and provider increases adherence to study protocols [18].

## Conclusions

Our study identified key themes pertinent to the design of cancer RCTs. Utilizing patient voices during the study design phase improves feasibility and provides opportunities for patient centered RCTs.

### Limitations

- One FG had only 1 patient, which did not allow for any patient-to-patient interaction and hampered the patient’s ability to construct social context for the discussion.
- We did not distinguish between the types of gynecologic cancer, which limit the applicability of the feasibility analysis to each category of gynecologic cancer.

### Electronic supplementary material

Below is the link to the electronic supplementary material.

Supplementary Material 1

## Data Availability

The datasets used and/or analyzed during the current study are available from the corresponding author on reasonable request.

## Abbreviations

FG: Focus groups
HFH: Henry Ford Health
IF: Intermittent fasting
iFIRE-C: Intermittent Fasting to Restrict Cancer
RCT: Randomized control trial
