# Supplementary material for: A patient perspective on applying intermittent fasting in gynecologic cancer
Source: BMC Res Notes. 2023 Aug 29;16:190. doi: 10.1186/s13104-023-06453-5 (PMC10466878; doi:10.1186/s13104-023-06453-5)
Supplement: Supplementary file 1 — Supplementary Material 1 [file 13104_2023_6453_MOESM1_ESM.pdf]

iFIRE-C Focus Group Survey

Research Study Recruitment Methodologies

1. What method of recruitment would you be more receptive too?

- ☐ Social media
- ☐ In person recruitment (from physician or study staff)
- ☐ Calls
- ☐ Texts
- ☐ Email Flyer
- ☐ Other (please specify)

2. Would you be more likely to take part in the study if approached by a trained/certified medical professional (example: nurse coordinator)?

- ◯ Yes
- ◯ No
- ◯ I'm not sure

Research Study Communications

3. What is your preferred method of communication?

- ◯ Call
- ◯ Email
- ◯ Text
- ◯ Letters in the mail

4. How often would you like a call/message from a study staff member?

- ◯ Daily
- ◯ Once a week
- ◯ Twice a month
- ◯ Other (please specify)

5. Would you like scheduled weekly check-ins with clinic staff to ask questions, provide feedback?

- ◯ Yes
- ◯ No
- ◯ I'm not sure

6. Would you like scheduled check-ins with a registered dietician?

- ◯ Yes
- ◯ No
- ◯ I'm not sure

Eating habits, lifestyle, and food preferences

7. Are you currently taking any appetite suppressants or stimulants (any agent that reduces desire for food)?

- ◯ Yes
- ◯ No

If yes, please share:

8. On Average, how many days a week do you exercise for at least 30 minutes?

- ◯ None
- ◯ 1-2 days
- ◯ 3-4 days
- ◯ 5-6 days
- ◯ Everyday

9. How many times do you eat per day (including snacking)?

- ◯ Once a day
- ◯ 2-3 times a day
- ◯ 4-5 times a day
- ◯ 6 or more times a day

Chemotherapy

10. While on Chemo, do you suffer from nausea?

- ◯ Yes
- ◯ No

11. In general, do you feel hungrier than usual on chemo or less?

- ◯ Hungrier than usual
- ◯ Less hungry than usual
- ◯ No change in appetite

12. How much are you usually able to eat during chemo?

- ◯ As much as I want, no changes during chemo
- ◯ Moderate: I am able to eat slightly less during chemo
- ◯ Much less: I have trouble eating during chemo
- ◯ Other (please specify)

13. What have been some of the side effects from Chemo on your appetite?

Dieting/Fasting

Fasting is defined as abstaining from all or some kinds of food or drink during certain periods of time.

14. Have you ever fasted before?

- ◯ Yes
- ◯ No

15. Would your work/lifestyle interfere with sticking to a fasting diet?

- ◯ Yes
- ◯ No
- ◯ I'm not sure

16. If you were told that you could have as much water, coffee, or herbal tea (no sugar or artificial sweeteners) during your fasting periods would you be more likely to follow to a fasting diet?

- ◯ Yes
- ◯ No
- ◯ I'm not sure

17. How do you typically feel when you are hungry?

- ☐ Aggravated
- ☐ Irritated
- ☐ Lethargic
- ☐ Stressed
- ☐ Irritable
- ☐ Other (please specify)

18. If you exercise daily, do you think fasting will interfere?

- ◯ Yes
- ◯ No
- ◯ I'm not sure

19. Would your loved ones be supportive of a fasting diet?

- ◯ Yes
- ◯ No
- ◯ I'm not sure

20. Would you be open to enrolling in a study as a cancer patient that would ask that you fast/restrict eating for potentially 12 hours or more per day over a span of 12 weeks or more?

- Yes
- No
- Need more information
